# Clinical response to varying pollen exposure in allergic rhinitis in children in The Netherlands

**DOI:** 10.1186/s12887-023-04021-1

**Published:** 2023-05-24

**Authors:** Ellen Tameeris, Arthur M. Bohnen, Patrick J. E. Bindels, Gijs Elshout

**Affiliations:** Department of General Practice, Erasmus Medical Center Rotterdam, Doctor Molewaterplein 40, 3066GD Rotterdam, The Netherlands

**Keywords:** Allergic Rhinitis, Pollen counts, Pollen season definition, Symptom variation, Children

## Abstract

**Background:**

Allergic rhinitis (AR) affects 10–15% of children. Symptoms in seasonal AR are influenced by pollen exposure. Pollen counts vary throughout the pollen season and therefore, symptom severity fluctuates. This study investigates the correlation between pollen concentration and symptom load in children with AR in The Netherlands.

**Methods:**

A secondary analysis was performed in a study determining the most effective treatment for children with seasonal AR. Symptoms were measured during three months in 2013 and 2014 using a daily symptom diary. The pollen concentration was measured with a Hirst type volumetric spore trap sampler. A correlation coefficient was calculated for the correlation between the pollen concentration and the mean daily symptom score. The study protocol was approved by the medical ethical review committee of the Erasmus MC and is incorporated in the International Clinical Trials Registry Platform (EUCTR2012-001,591–11-NL).

**Results:**

In 2014, the correlation coefficient for birch pollen concentration and symptom score was 0.423 (*p* = 0.000). The correlation coefficient for grass pollen concentration and symptom score was 0.413 (*p* = 0.000) and 0.655 (*p* = 0.000) in 2013 and 2014, respectively. A delayed correlation between the birch pollen concentration and the symptom scores was seen up to two days after the pollen measurement (0.151, *p* = 0.031). For grass pollen this effect lasted up to three days after the pollen measurement (0.194, *p* = 0.000).

**Conclusion:**

We found comparable correlations between symptom score and pollen concentration as found by EAACI. Birch and grass pollen have an elongated influence on symptom score of several days. This implies patients need to continue on demand medication longer after a measured pollen peak.

**Supplementary Information:**

The online version contains supplementary material available at 10.1186/s12887-023-04021-1.

## Introduction

Allergic rhinitis (AR) is a common disorder of the upper airways [1]. 10–15% of children in the UK are affected [2]. Symptoms in seasonal AR can be nasal or ocular and arise when patients become sensitized to aeroallergens such as pollen.

Antihistamines (AH) or intranasal corticosteroids (INCS) [1] are safe and an effective treatment options for children with AR [3]. Standard treatment of allergic rhinitis is continuous use of INCS throughout the pollen season [1, 4]. However, adherence to this advice is limited [5]. Intermittent use of intranasal corticosteroids has proven to be equally effective in treating AR symptoms, hereby limiting total steroid intake [3, 6]. This limitation in steroid intake could minimise side-effects.

AR symptoms are directly influenced by patients’ exposure to pollen [7]. Pollen counts vary throughout the pollen season (PS), dependent on weather conditions and flowering period of plants [8]. The European Academy of Allergy and Clinical Immunology (EAACI) formed a definition of PS and peak pollen periods (PP) [9]. A correlation between birch and grass pollen concentration and symptom score was found for adult patients with AR in European countries during the defined PS an PP [10]. Predictions of pollen concentrations can help individuals to adapt their medication use to expected pollen concentration and symptom load. Additionally, activities could be adjusted. The definition for PS and PP has not been verified in children. This study aims to investigate the correlation between PS, pollen concentration and symptom load in children with AR.

## Methods

### Patients

A secondary analysis was performed in a study determining the most effective treatment for children with seasonal AR, for details see Wartna et al [3]. In this single-blinded trial children aged 6–18 years were randomized (after signing informed consent) into three treatment groups during the PS of 2013 and 2014. The treatment options were: INCS (fluticasone) on a daily basis, INCS on demand and AH (levocetirizine) on demand. Patients with an International Classification of Primary Care code for AR or a previous prescription for AR-medication registered in the electronic medical file were selected. Patients needed a mean total symptom score (TSS) over the last season high enough to be classified as symptomatic (7 or more out of 21 points). The screening score consisted of recalling four nasal and three ocular symptoms from the previous season on a 0 to 3 scale (Appendix I). A positive radioallergosorbent test (RAST) for grass pollen was required. The study protocol was approved by the medical ethical review committee of the Erasmus MC and is incorporated in the International Clinical Trials Registry Platform (EUCTR2012-001,591–11-NL).

### Symptom measurement

Included children had to rate their ocular and nasal symptoms daily during the PS from April until July in 2013 or 2014. This symptom diary was previously used in a study on the effectiveness of sublingual immunotherapy in children with AR [11]. Symptoms were rated from 0 (no burden) to 3 (very high burden) (Appendix II). Symptoms scored were sneezing, nasal itch, congestion or rhinorrhea and ocular itch, redness or teary eyes and wheezing, shortness of breath and nighttime coughing. The TSS range was 0 to 30. Available symptom scores were averaged to a mean daily TSS.

The summer holidays started at July 1^st^. During this period participants went on holiday and were residing further away from the pollen counting station. Data between July 1^st^ and July 31^st^ in both observed years were not used in the correlation analysis.

### Pollen counts

Pollen concentration was determined using pollen counts from the Leiden University Medical Center (LUMC). The LUMC uses a Hirst type volumetric spore trap sampler to assemble daily pollen from the air. The assembled pollen samples are then microscopically identified. Pollen counts are reported daily as grains/m3 [12].

PS was defined following the criteria stated by EAACI. Birch PS starts at the first of five consecutive days with the birch pollen concentration ≥ 10 grains/m^3^ and if the sum of the pollen count on these five days is ≥ 100 grains/m^3^. The season ends at the last day of five days with a pollen concentration of ≥ 10 grains/m^3^. Grass PS starts on the first day of five consecutive days on which the grass pollen concentration rises to or above 3 grains/m^3^, and if the sum of the pollen count on these five days is ≥ 30 grains/m^3^. The season ends on the last day of a series of five consecutive days with a pollen concentration of ≥ 3 grains/m^3^, with a sum of these days of ≥ 30 grains/m^3^. Peak pollen concentrations were defined as a pollen count > 100 grains/m^3^ for at least three consecutive days for birch pollen and > 50 grains/m^3^ for at least three consecutive days for grass pollen [9].

### Statistical analysis

Spearman correlation coefficients between daily pollen concentrations and mean TSS were calculated [13]. For birch pollen correlations only birch sensitive patients were analysed and the period of analysed measurements included days with grass pollen load < 5 grains/m3. For grass pollen correlations the period included days with birch pollen load < 5 grains/m3. As the relationship between pollen concentration and symptom score was nonparametric, a Spearman correlation coefficient was calculated.

To determine a possible delayed effect of birch pollen concentration on TSS, the subsequent days were investigated using lagged pollen concentration. The pollen concentrations were compared to TSS one to four days later.

A sub analysis was performed for the correlation between birch pollen concentration and TSS in patients with birch pollen polysensitisation. In addition, a sub analysis was performed for peak pollen concentration, type of symptoms (nasal or ocular), children/adolescents and distance from the study subjects to the pollen counting station. The postal codes of the patients’ address were collected to investigate if the distance from the pollen counting site influenced the correlation. Differences in baseline characteristics were calculated using the Chi-square test for categorical variables and the ANOVA test for continuous variables [14].

Missing data were analysed and imputed using multiple imputation. Analysis on the pooled data was reported. Statistical analyses were performed using SPSS statistics version 25.

## Results

### Baseline characteristics

One hundred fifty children were included in the PS of 2013 and 2014. The participants were randomised to three treatment groups: INCS daily (*N* = 50), INCS on demand (*N* = 52) and AH on demand (*N* = 48). 52% of the participants were boys (*N* = 78), mean age was 11.6 years. There were no significant baseline differences in patient characteristics (Table 1). Of the birch sensitised patients 69.4% were adolescents (*p* = 0.021). 16.7% of the diary entries was missing. There was no significant difference in the percentage of symptom free days between the randomisation groups [3].

**Table 1:** Baseline characteristics per cohort

	**2013 (*n* = 51)**	**2014 (*n* = 99)**	***P*-value**
Sex (boys), *N (%)*	24 (47.1)	54 (54.5)	0.385
Age (years), *mean (SD)*	11.85 (0.54)	11.57 (0.31)	0.634
Asthma, *N (%)*	8 (15.7)	15 (15.1)	0.931
Grass sensitisation, *N (%)*	51 (100)	99 (100)	0.471
Birch sensitisation, *N (%)*	29 (56.9)	60 (60.6)	0.658
HDM sensitisation, *N (%)*	26 (51.0)	49 (49.5)	0.863
Monosensitisation, *N (%)*	11 (21.6)	24 (24.2)	0.712
Current use of secondary care for AR, *N (%)*	2 (3.9)	12 (12.1)	0.127
Grass sensitisation class > 2, *N (%)*	38 (74.5)	59 (59.6)	0.043
Birch sensitisation class > 2, *N (%)*	29 (56.9)	62 (62.7)	0.367
Retrospective symptom score, *mean (SD)*	12.31 (3.10)	11.93 (3.18)	0.477
Baseline symptom score	11.47 (4.10)	10.34 (4.30)	0.319

Mean distance from the pollen counting station was 22.26 kms (KM) (SD 10.59).

### Pollen seasons and peaks

The birch PS in 2013 lasted from April 23^rd^ until May 13^th^, the grass PS lasted from May 27^th^ until July 31^st^. In 2014 the birch PS lasted from April 1^st^ until April 23^rd^, the grass season lasted from May 15^th^ until June 28^th^. Mean grass pollen concentration differed significantly between 2013 and 2014 (Table 2). In 2013 birch PP concentrations occurred from May 2^nd^ until May 4^th^ grass PP did not occur during the study period. In 2014 a birch PP occurred from April 2^nd^ until April 4^th^, grass PP occurred from June 1^st^ until June 3^rd^ and from June 6^th^ until June 9^th^. The PP were too short for analysis of correlation.

**Table 2:** Pollen concentrations per year (grains/m3)

	**Min**	**Max**	**Mean**	**SD**
2013				
Birch Pollen	0.00	305.00	18.84	55.76
Grass Pollen	0.00	171.00	14.11*	29.68
2014				
Birch Pollen	0.00	1203.00	22.27	114.15
Grass Pollen	0.00	168.00	19.16*	28.64

^*^ *p* < 0.05 for 2013 compared to 2014

### Symptom scores

**Fig. 1:**
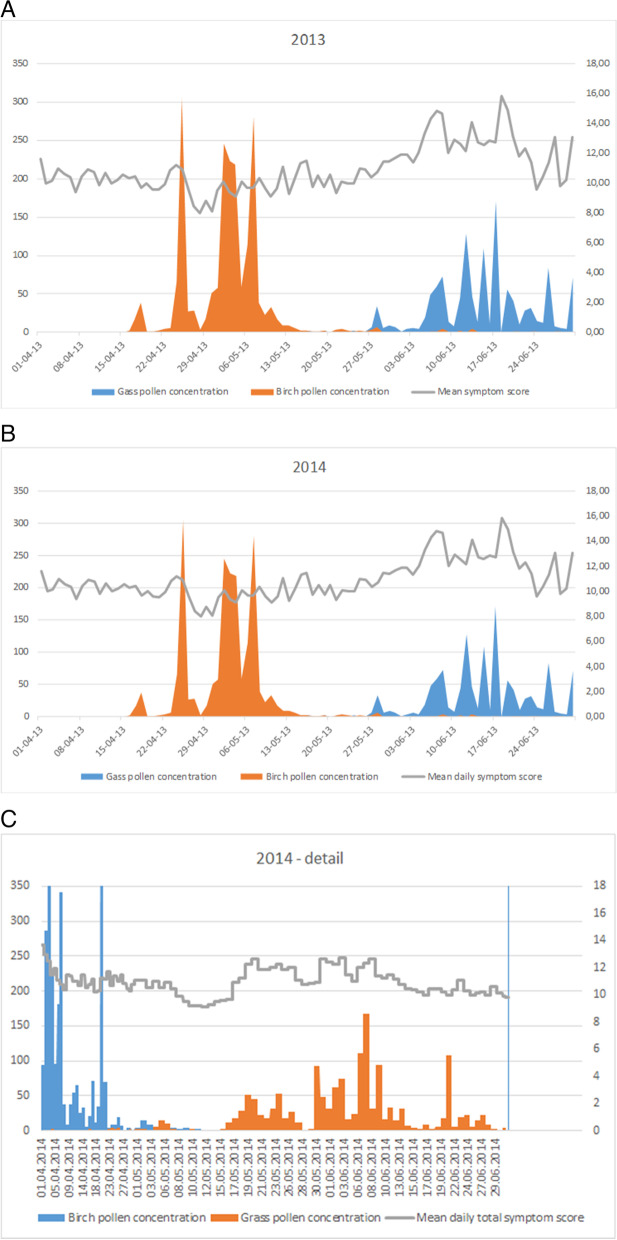
**A**—graphical presentation of pollen concentration & symptom score in 2013. **B**—graphical presentation of pollen concentration & symptom score in 2014. **C**—detail of pollen concentration & symptom score 2014

The mean TSS in 2013 was 10.97 (SD 1.67), the mean nasal symptom score (NSS) was 6.82 (SD 1.01), the mean ocular symptom score (OSS) was 4.15 (SD 0.82). In 2014, the mean TSS was 10.72 (SD 1.07), the mean NSS was 6.31 (SD 0.65) and the mean OSS was 4.41 (SD 0.47). Mean TSS, NSS and OSS differed significantly between the two cohorts (*p* = 0.001). However, this difference is probably not clinically relevant. Pollen concentration levels and mean TSS per PS are graphically represented in Fig. 1A-C.

#### Correlation between pollen concentration and symptom scores

The correlation coefficients between TSS and birch pollen concentration in birch sensitive patients during birch PS in 2013 were not significant. In 2014, this correlation was 0.423 (*p* = 0.000) (Table 3).

**Table 3:** Correlation between symptom score and birch pollen concentration for birch pollen sensitive patients

	**2013**			**2014**		
**Variable**	**Spearman’s Rho**	**95% CI**	***P*-value**	**Spearman’s Rho**	**95% CI**	***P*-value**
Daily score mean	0.248	0.110–0.377	0.201	0.421	0.347–0.490	0.000
Daily Ocular score mean	0.176	0.036–0.309	0.455	0.329	0.251–0.403	0.002
Daily Nasal score mean	0.210	0.071–0.341	0.110	0.412	0.337–0.482	0.000

Measurements performed during grass pollen load < 5 grains/m3

There is a stronger correlation between TSS and birch pollen concentration in birch sensitive adolescents compared to children < 10 years (Table 4).There was no difference in correlation coefficients between the treatment groups (Appendix II).

**Table 4:** Spearman correlation coefficients between pollen counts and the mean symptom scores in children < 10 years and adolescents

			**2013**				**2014**		
	**Variable**	**Birch season**	**95% confidence interval (CI)**	**Grass season**	**95% CI**	**Birch season**	**95% CI**	**Grass season**	**95% CI**
Children < 10 years	Mean total symptom score	0.175	-0.249–0.543	0.462*	0.256–0.628	0.380*	0.225–0.516	0.670*	0.630–0.706
	Mean ocular symptom score	0.076	-0.243–0.547	0.413*	0.201–0.588	0.312*	0.207–0.501	0.630*	0.587–0.669
	Mean nasal symptom score	0.181	-0.388–0.466	0.456*	0.225–0.623	0.363*	0.153–0.456	0.690*	0.652–0.724
Adolescents	Mean total symptom score	0.243	0.086–0.388	0.408*	0.315–0.493	0.423*	0.334–0.505	0.653	0.629–0.675
	Mean ocular symptom score	0.204	0.015–0.323	0.389*	0.274–0.457	0.414*	0.324–0.496	0.674	0.589–0.638
	Mean nasal symptom score	0.173	0.046–0.352	0.369*	0.295–0.475	0.330*	0.236–0.418	0.614	0.651–0.695

^*^ *p* < 0.05

The correlation coefficients for lagged birch pollen concentration (the pollen concentration of one and two days prior to the recorded symptom score) became weaker and non-significant after two days. The correlation coefficient between grass pollen concentration and mean TSS during the grass PS in 2013 was 0.413 (*p* = 0.000). In 2014 this correlation was 0.655 (*p* = 0.000). The correlation coefficients for lagged grass pollen concentration decreased, but remained significant until three days after the measured pollen concentration (Table 5).

**Table 5:** Correlation between symptom score and lagged grass pollen concentration (number of days) during grass pollen season (birch pollen load < 5 grains/m3)

**Variabele**	**Spearman’s Rho (daily score)**	***P*-value**	**Spearman’s Rho (Ocular score)**	***P*-value**	**Spearman’s Rho (Nasal score)**	***P*-value**
**2013**						
Lag grass 1 day	0.321	0.000	0.350	0.000	0.232	0.000
Lag grass 2 days	0.147	0.000	0.179	0.000	0.078	0.000
Lag grass 3 days	0.194	0.000	0.250	0.000	0.031	0.129
**2014**						
Lag grass 1 day	0.626	0.000	0.581	0.000	0.627	0.000
Lag grass 2 days	0.490	0.000	0.488	0.000	0.473	0.000
Lag grass 3 days	0.296	0.000	0.303	0.000	0.292	0.000

The correlation coefficient between mean TSS and the distance from the study subject’s home address to the pollen measuring station was very small and not significant. There was no significant difference in correlation coefficient between subjects living within or further than 30 km from the pollen measuring station (Table 6).

**Table 6:** Correlation between mean total symptom score and grass or birch pollen concentration for children living < 30 KM and > 30 KM from the pollen measuring station

Variable	Spearman’s Rho	95% Confidence interval	*P*-value
< 30KM Birch pollen	0.109	0.084–0.133	0.000
> 30KM Birch pollen	0.185	0.127–0.242	0.000
< 30KM Grass pollen	0.367	0.345–0.389	0.000
> 30KM Grass pollen	0.339	0.284–0.392	0.000

## Discussion

This study confirms the correlation between birch and grass pollen concentration and TSS in children and adolescents with AR in The Netherlands. In addition, it confirms a delayed effect of several days, which implies that patients need to take on demand medication for more days after a registered PP. We found variable correlations over the two studied seasons, which can be explained by seasonal variations in pollen concentration.

There is a stronger correlation between TSS and birch pollen concentration in birch sensitive adolescents compared to children < 10 years. This result could be influenced by the higher prevalence of birch and grass polysensitisation among adolescents [15]. The correlation coefficients between TSS and grass pollen concentration did not differ significantly between children and adolescents.

When analysing lagged correlation between mean TSS and pollen concentration, the correlation coefficient decreases, but remains significant for pollen concentrations from two (birch pollen) to three (grass pollen) days prior to the diary entries. This delayed effect can implicate that pollen concentrations might be of importance not only on the current day, but also subsequent days. The delayed influence of pollen concentration could be explained by pollen grains being trapped inside buildings, or a chronic state of inflammation of the nasal mucosa [16, 17]. This implies that patients might need to continue their on demand medication up to three days after a measured PP.

Better alignment between TSS and treatment can lead to improved symptom control, quality of life and decrease in steroid intake by optimising as-needed intake of medication. The EAACI proposed definitions for PS and peak pollen concentration for use in clinical trials [10]. This study confirms the correlation of the proposed pollen concentration levels to define grass and birch PS for children and adolescents with AR in The Netherlands.

Karatzas et al. confirmed the definitions of PS and peak pollen period as proposed in the EAACI position paper for two German areas [18]. During the study, pollen concentrations correlated to the observed rhinitis symptom scores. Pfaar et al. also confirmed these findings for regions in Austria, Finland and France [10]. Both studies are based on open-access hay-fever diaries, which leaves exact characteristics of the studied population unknown. The correlation coefficients between birch pollen concentration and mean TSS found in our study are weaker. Only part of our study population is sensitised to birch pollen. In addition, the birch pollen concentration in 2013 is too low to find a significant correlation between TSS and pollen concentration. The correlation between symptom score and grass pollen concentration is comparable to the levels of correlation found by Karatzas and Pfaar [10, 18].

The mean birch and grass pollen concentration differed significantly between the two observed PS. These findings could well be explained by annual variation in weather conditions and environmental factors and are therefore not surprising. These findings are also supported by the significant difference in mean TSS, differences in correlation coefficients between the two years and lack of a statistical significant correlation between birch pollen concentration and TSS in 2013. The relatively low levels of pollen concentration in 2013 resulted in a weaker correlation between pollen season and symptom score, and pollen concentration and symptom score.

The participants in this study were asked to fill in a symptom diary every day for a period of three months. Symptom scores were also reported in times of low pollen exposure. This gives a more fair insight in the correlation between pollen concentration and TSS compared to studies based on voluntary input of AR symptoms. The fact that participants did not only fill in the diaries in case of (severe) symptoms could explain the lower correlation found in this study compared to the results found by Karatzas & Pfaar [10, 18].

Participants lived a maximum of 66 km away from the pollen measuring location. The area is variable in environment, from farmland to urban, which could have resulted in local pollen concentrations that are different from the used measurements. The LUMC pollen measuring station is the nearest station to the study participants and is used as one of the Dutch reference stations to inform patients on PP. In previous research, pollen concentration estimations have been found reliable up to 30 km from the measuring station [19]. 29.4% of the study participants lived more than 30 km away from the station. However, we found no significant difference in the correlation between pollen concentration and mean TSS among study participants living within 30 km or further than 30 km from the pollen measuring station.

Study participants were randomised into three treatment groups: INCS continuous, INCS on demand and AH on demand. The amount of symptom free days did not differ significantly between the three treatment groups [3]. These results implicate that the correlation between symptom score and pollen concentration holds regardless of treatment for AR.

Our results enable the use of the proposed definition for PS and PP for both clinical trials and daily clinical routine in The Netherlands for children and adolescents with AR. Measured and predicted pollen concentrations can be used to predict an increase or decrease in symptom score and guide medication intake in children with AR. Weather and pollen concentration forecasts, improved by better knowledge of PS and peaks, can be used to anticipate outside activities of AR patients. As-needed use provides half of the corticosteroid exposure compared to regular, continuous treatment [6]. In addition, adherence to prescription of continuous use of INCS is often poor: 40–71.5% [20]. Adding as-needed medication use to the treatment options in shared decision making might improve adherence. In case of a PP, as-needed medication may be useful on the following 2–3 days due to a delayed development of nasal and ocular symptoms after pollen exposure. Even if locally expected pollen exposure is low in these days.

## Supplementary Information

**Additional file 1: eAppendix 1.** Screening questionnaire.** eAppendix 2.** symptom diary.** eAppendix 3.** Pooled Spearman correlation coefficients.** eAppendix 4.** Abbreviations used.

## Data Availability

The datasets used and/or analysed during the current study are available from the corresponding author on reasonable request.

## Abbreviations used

AR: Allergic Rhinitis
AH: Antihistamine
INCS: Intranasal Corticosteroids
EAACI: European Academy of Allergy and Clinical Immunology
LUMC: Leiden University medical Centre
KM: Kilometres
TSS: Total Symptom Score
NSS: Nasal Symptom Score
OSS: Ocular Symptom Score
PS: Pollen season
PP: Pollen peak
