# Supplementary material for: Clinical response to varying pollen exposure in allergic rhinitis in children in The Netherlands
Source: BMC Pediatr. 2023 May 24;23:258. doi: 10.1186/s12887-023-04021-1 (PMC10207782; doi:10.1186/s12887-023-04021-1)
Supplement: Supplementary file 1 — Additional file 1: eAppendix 1. Screening questionnaire. eAppendix 2. symptom diary. eAppendix 3. Pooled Spearman correlation coefficients. eAppendix 4. Abbreviations used. [file 12887_2023_4021_MOESM1_ESM.docx]

**Appendixes**

**eAppendix 1 – Screening questionnaire**

This questionnaire was sent to patients meeting inclusion criteria in their patient file. Eligibility was further investigated using the symptom score for the past pollen season. A minimum of 7 out of 21 points was required. The original questionnaire was presented in Dutch.

1. Does your child have hay fever (grass or tree pollen allergy)?

Answer options: yes/no/unknown.

1. Did your child experience hay fever symptoms during last hay fever season (March – August)?

Answer options: yes/no/unknown.

1. Did your child experience one of the following complaints during last hay fever season (March – August)?
   1. Sneezing
   2. Itching nose
   3. Runny nose
   4. Nasal congestion
   5. Teary eyes
   6. Itching eyes
   7. Red eyes

Answer options: 0 – none, 1 – little, 2 – much, 3 – very much

**eAppendix 2 – symptom diary**

This questionnaire was sent to the participants on a daily base. The diaries were filled in digitally, with some exceptions filling in an analogue copy of the digital diary. Patients received a daily reminder to fill in the diary by e-mail and text message (SMS). The original questionnaire was presented in Dutch.

How much do you experience the following symptoms?

1. Sneezing
2. Itchy nose
3. Runny nose
4. Nasal congestion
5. Teary eyes
6. Itchy eyes
7. Red eyes
8. Wheezing
9. Dry cough at night
10. Shortness of breath

Answer options: 0 – none, 1 – little, 2 – much, 3 – very much

1. Did you use your medication today?

Answer options: yes/no

1. Did you use your emergency medication?

Answer options: yes, nasal spray; yes, eyedrops; yes, else, namely: …; no

1. Did you use other medication (regardless of reason)?

Open question

1. Did you experience other complaints than mentioned above?

Open question

**eAppendix 3 – Pooled Spearman correlation coefficients**

Correlation between symptom score and lagged birch pollen concentration (number of days) for birch pollen sensitive patients during birch pollen season (grass pollen load <5 grains/m3):

|  | **2013** | | **2014** | |
| --- | --- | --- | --- | --- |
| **Variable** | **Spearman’s Rho** | **P-value** | **Spearman’s Rho** | **P-value** |
| Lag birch 1 day | 0.025 | 0.896 | 0.151 | 0.031 |
| Lag birch 2 days | -0.013 | 0.91 | 0.073 | 0.177 |

Correlation between symptom score and grass pollen concentration during grass pollen season (birch pollen load <5 grains/m3):

|  | **2013** | | | **2014** | | |
| --- | --- | --- | --- | --- | --- | --- |
| **Variable** | **Spearman’s Rho** | **95% CI** | **P-value** | **Spearman’s Rho** | **95% CI** | **P-value** |
| Daily score mean | 0.413 | 0.324-0.495 | 0.002 | 0.655 | 0.635-0.674 | 0.000 |
| Daily Ocular score mean | 0.373 | 0.282-0.458 | 0.003 | 0.617 | 0.596-0.638 | 0.000 |
| Daily Nasal score mean | 0.394 | 0.304-0.477 | 0.000 | 0.676 | 0.657-0.694 | 0.000 |

Correlation between symptom score and pollen concentrations per intervention group:

|  | **Birch pollen** |  | **Grass pollen** |  |
| --- | --- | --- | --- | --- |
|  | **Spearman correlation** | **Sig.** | **Spearman correlation** | **Sig.** |
| INCS continuous | 0.420 | <0.001 | 0.554 | 0.000 |
| INCS on demand | 0.428 | <0.001 | 0.499 | 0.000 |
| AH on demand | 0.263 | 0.018 | 0.463 | <0.001 |

**eAppendix 4 – Abbreviations used**

AR – Allergic Rhinitis

AH – Antihistamine

INCS – Intranasal Corticosteroids

EAACI – European Academy of Allergy and Clinical Immunology

LUMC – Leiden University medical Centre

KM – Kilometres

TSS – Total Symptom Score

NSS – Nasal Symptom Score

OSS – Ocular Symptom Score

PS – Pollen season

PP – Pollen peak
